# Sensitive imaging of actinide materials in shielded radioactive waste

**DOI:** 10.1038/s41598-024-78027-9

**Published:** 2024-11-05

**Authors:** Jana Vasiljević, Vivian Peters, Anders Puranen, Bo Cederwall

**Affiliations:** 1 Department of Physics, KTH Royal Institute of Technology, 106 91 Stockholm, Sweden; 2 AB Svafo, 611 23 Nyköping, Sweden

**Keywords:** Physics, Nuclear physics, Experimental nuclear physics

## Abstract

This paper reports on the development of a method for enhanced non-destructive assay (NDA) of radioactive waste using the novel technique neutron-gamma emission tomography (NGET). The technique relies on the detection of correlated fast neutrons and gamma rays emitted in spontaneous or induced fission. It is based on fast organic scintillators and enables sensitive detection and three-dimensional (3D) localization of the fission events. The technique is passive and does not require moving components. In this work, we apply the NGET technique to the category of radioactive waste which is often referred to as historic or legacy waste. This can include mixed wastes encased in shielded containers many decades ago, before the advent of detailed waste description criteria. These low or intermediate level wastes are often associated with lacking, limited or conflicting documentation. This poses a challenge when assigning the waste to the proper disposal route as well as in deciding whether the waste needs to undergo sorting and conditioning to fulfil waste acceptance criteria both with regards to safe interim storage and to its ultimate disposal. Actinides, such as isotopes of uranium and plutonium, with their typically long half-lives and decay chains are of special interest in this regard since they may challenge the long-term safety assessment in repositories predicated on mainly shorter half-life radionuclides if undetected. Accurate identification and localisation of actinides is also important from a safeguards perspective, especially since they are generally difficult to detect and localise by established passive means due to their relatively weak radiation emissions, in particular in shielded containments and in the presence of strong radiation fields from other radioactive materials. In this paper we present findings of measurements on shielded containments of long-lived radioactive waste performed at the Studsvik site in Sweden, as well as measurements on a laboratory assembly simulating a grouted waste drum. Similarities and differences between the novel NGET technique and a commercially available gamma imaging system are also briefly discussed.

## Introduction

Radioactive materials are produced or used in different applications that benefit humankind, such as production of electricity, medicine, academic research and industry. From all these applications, waste is produced with different composition, radioactivity levels and half-lives. Keeping the public and the environment safe from radiation exposure due to such waste is a societal task of great importance. Furthermore, for special nuclear materials (SNM), the risk of proliferation of nuclear weapons and nuclear terrorism constitute additional threats that has led to an extensive international regulatory framework coordinated by the International Atomic Energy Agency (IAEA). In December 2016 there was an estimated 38 million m^3^ of solid radioactive waste, based on information provided by 38 Member States of the IAEA^1^. Since spent nuclear fuel (SNF) is often well characterized, the main challenge lies in verification of operator declarations for nuclear safeguards purposes as governed by the IAEA, and secure handling either in terms of reprocessing or safe interim storage for a long period of time pending disposal in final repositories. There are, additionally, substantial quantities of low- and intermediate-level radioactive waste that is classified as long-lived which consequently also needs safe long-term disposal. A significant fraction of the radionuclide inventory in these materials comes from early nuclear research, including defense-related research activities, but it also contains long-lived radioactive wastes from the industry and the healthcare sector. This type of waste poses special challenges in its characterization due to its frequently poor or missing documentation and inhomogeneous composition. The materials are sometimes stored in difficult to open sealed containers with significant amounts of shielding. The handling of such radioactive waste therefore requires special measurement capabilities to enable its accurate characterization.

Although destructive analysis techniques, e.g. based on sampling and radiochemical analysis, may provide a more complete and accurate characterization of the radionuclide inventory^2^, they require extraction of the radioactive material from its containment and can only access a small part of the radioactive contents at a time. NDA methods, being often less expensive, less time consuming and requiring less radiation safety measures than destructive methods, can provide assessment of the full contents of a sealed waste container and are therefore suitable for initial characterization. Improvements in NDA techniques that provide detection and accurate localization of the radioactive material, in particular actinides, without breaching the external shielding of the waste matrix would therefore constitute a major advance in characterization techniques, taking into account also requirements of nuclear materials control, as well as providing guidance and a potential reduction in the need for intrusive assay techniques.

There are several different passive and active NDA techniques already implemented for radioactive waste characterization, such as gamma-ray spectroscopy, neutron counting, gamma-ray emission/transmission imaging, neutron activation and others^2–4^. Passive NDA techniques are typically easier to implement and faster, as they require no external photon or neutron source. Actinides predominantly emit low energy gamma rays, which are easily shielded in the complex waste containers or self-attenuated. This makes passive gamma-ray detection challenging, especially if it is applied in environments with elevated gamma-ray background radiation levels. Neutron emission is a characteristic signature of actinides as such materials invariably emit fast neutrons at rates depending strongly on the particular isotope. In general, combined fast-neutron and gamma-ray detection^5–10^ is appealing for detecting the presence of actinides in shielded radioactive waste containers.

The nuclear decommissioning and waste management company AB Svafo is charged with interim storage and characterization of the Swedish Legacy Waste (SLW) from nuclear research and other, in particular, nuclear facilities^11–13^. This radioactive waste often has varied origin and mixed composition, ranging from waste from nuclear research reactors to non-nuclear radioactive waste from medical, industrial and research applications. The waste is currently stored in approximately ten thousand drums awaiting further characterization before potentially being opened and their contents separated into different categories in preparation for a final geological repository. The total activity of each shielded waste drum has typically been limited so that the dose rate outside the wall is less than around 2 μSv/h. Further details on the SLW program can be found in^14^. Larger quantities of radioactive waste with similar characteristics are likely present in temporary storage worldwide, making it a global concern. The characterization task is challenging due to lack of or inaccurate documentation of the radioactive contents. Adding to this complexity, the waste acceptance criteria for disposal in Sweden have changed from during the waste generation period (from the 1960s) until now which requires careful characterization and classification of each waste drum. A considerable fraction of the waste drums have similar form factors. They are typically of 200 to 300 l volume enclosing an inner drum with the waste form and typically a 5 cm concrete lining. The thickness of the concrete may however reach tens of cm in some cases. Additional shielding such as lead or rebar may also be present. The waste may contain SNM under safeguards in various forms, such as uranium and plutonium in solid form or liquid solutions containing these and other actinide elements. In this work we present detailed results from applying the neutron-gamma emission tomography (NGET) technique^15^ for examining radioactive waste^16^. Results from laboratory measurements with radioactive sources placed inside a mockup waste drum assembly are also presented. A commercially available H420 gamma imager from H3D was also employed at the laboratory test setup for a brief comparison of the techniques. The detection system used for NGET imaging in the present work is based on large organic scintillators and is highly sensitive to fast neutrons and gamma rays. It is therefore also suitable for passive fast-neutron and low-resolution gamma counting employing standard techniques (not discussed here).

## Methods

### Experimental setup

**Fig. 1:**
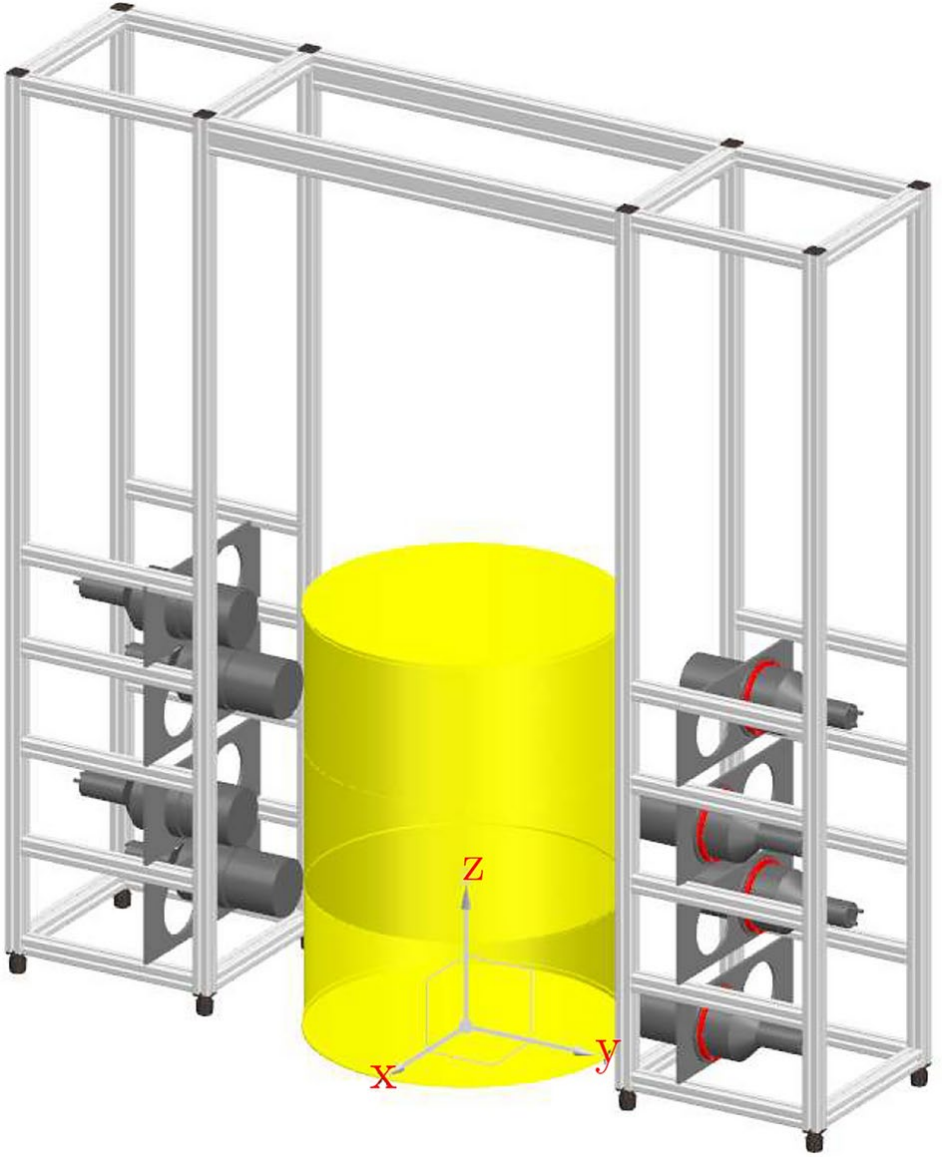
Schematic drawing of the experimental configuration used for the laboratory measurements of the mockup waste drum assembly. The detector assemblies covered approximately a 100 cm vertical range. The horizontal distance between the detector front faces was 100 cm. See text for further details.

We here present results from measurements using an array of eight 127 mm diameter by 127 mm height cylindrical detector cells containing the liquid organic scintillator EJ-309^17^. The scintillation light pulses were read out by Hamamatsu R1250 photomultiplier tubes (PMT)^18^ for which the anode pulses were registered using an eight-channel CAEN DT5730 digitizer board^19^ featuring 2-Vpp dynamic range, 14-bit resolution, and 500-MHz sampling rate. The scintillator is sensitive to both gamma rays and fast neutrons and enables to distinguish between the different types of particles by means of pulse shape discrimination (PSD)^20^. Real-time PSD was accomplished by applying the charge comparison method^21,22^ to the digitized PMT signals. The organic scintillator detectors have fast timing properties, typically with a time resolution of the order of 1 ns^23^, which is an important feature for NGET imaging^15^. The radiation sensors were placed in a zig-zag pattern in two vertical pillars, with a 1 m distance between the front faces of detectors, covering approximately a 1 m height from the ground (Fig. 1). A detailed description of the detector system can be found in Ref.^22^. NGET image analysis based on iterative use of Bayesian inference was performed on data from laboratory measurements of a radioactive ^252^Cf spontaneous fission source positioned inside a mockup waste drum assembly as well as actual radioactive waste drums at the Svafo facility, Studsvik, Sweden.

### Data analysis

Bayes’ well-known theorem ^24^, provides a simple mathematical formula for calculating conditional probabilities and thereby enables an estimate for the probability of a hypothesis *H* to be true, given the occurrence of an event *E*:

1 $$\begin{aligned} P(H | E) = \frac{P(E | H) P(H)}{P(E)}, \end{aligned}$$

where *P*(*E*|*H*) is the probability of the event to occur given that the hypothesis is true, *P*(*H*) is the probability that the hypothesis is true and *P*(*E*) is the total probability that the event occurs. Here, an event, *E*, of interest is the detection of a gamma-neutron coincidence, i.e. the detection of one gamma ray and one neutron in two different detectors within the short time interval characteristic of particles emitted from the same fission event. The event is characterized by a time difference $\Delta t_{n \gamma }$ between the gamma-ray and neutron detection and a detected neutron-recoil energy, as measured from the light output from the scintillator, *L*. For each voxel in space, labeled by index *i*, we may then form the hypothesis $H_i$ that the event originated from the center of the voxel $\vec {r}_i$. The time difference can then be written as

2 $$\begin{aligned} \Delta t_{n \gamma } = t_{n} - t_{\gamma } = \frac{\left| \vec {r}_n - \vec {r}_i \right| }{v_{n}} - \frac{\left| \vec {r}_{\gamma } - \vec {r}_i \right| }{c}, \end{aligned}$$

where $\vec {r}_n$ and $\vec {r}_{\gamma }$ are the locations of the interaction points in the detectors registering the neutron and the gamma ray, respectively. However, no information on the interaction positions within the sensitive detector volume was available from the measured quantities. The primary interaction point is assumed to be the geometrical center for the detector registering a gamma ray. The neutron mean free path in the detector medium for typical kinetic energies relevant for emission in fission is up to a few centimeters^25^, i.e. significantly smaller than the dimensions of the sensitive volume in each detector element. In this work, the neutron flight path length for each event has been corrected assuming a neutron mean free path in the scintillator of 25 mm. For each voxel, *i*, the neutron kinetic energy is then derived as

3 $$\begin{aligned} T_{n}(\vec {r}_i) = m_n c^2 \left( \frac{1}{\sqrt{1-\left( \frac{v_n(\vec {r}_i)}{c}\right) ^2}} - 1\right) , \end{aligned}$$

where $v_n(\vec {r}_i)$ is derived from Eq. (2), *c* is the velocity of light, and $m_n$ is the neutron rest mass. The probability that a neutron with this kinetic energy is emitted from the source is then calculated from evaluated prompt fission neutron spectral data^26^. The predominant interaction of neutrons in the organic scintillator is elastic scattering on protons although scattering on nuclei of other elements in the detector material (primarily carbon) also needs to be taken into account. Each scatter transfers a portion of the neutron kinetic energy to the recoiling proton/nucleus and a fraction of this energy is converted into fluorescent light in the scintillator and read out as an electrical signal via a photomultiplier tube. The total charge of the resulting current pulse is integrated to provide a measure of the deposited energy. However, due to the nonlinear conversion of recoil energy into scintillation light and the stochastic nature of the scattering process, there is a large variation in light output at a given incident neutron energy. The probability that a neutron of a given kinetic energy produces a certain light output, the light-output response function $R(L|T_n)$, was calculated using the Monte Carlo code Geant4^27^ and the proton light-output function derived by Enqvist et al.^23^. The Monte Carlo simulations also take the measured detector energy resolution into account.

Given the hypothesis $H_i$ that the event originated from the center of the voxel $\vec {r}_i$, the probability of the event *E* to occur is calculated as

4 $$\begin{aligned} P(E | H_i) = \Phi (T_n) \, R(L|T_n)\delta T_n\frac{\Omega _n\Omega _{\gamma }}{(4\pi )^2}, \end{aligned}$$

where $\Phi (T_n)$ is the prompt fission neutron spectrum, $\delta T_n$ is the detector energy resolution, and $\Omega _{\gamma (n)}$ are the solid angles subtended by the detector registering the gamma ray (neutron) as seen from the voxel with index *i*. The solid angles were approximated by assuming that each detector element had a spherical geometry. For objects placed well inside an array of identical detector elements, as in the present work, the product of the solid angles subtended by the detector registering the fast neutron and the detector registering the coincident gamma ray is approximately constant as a function of the position between the detectors. This is a general feature of detector systems with identical detector elements operated in coincidence mode. The approximation was made in previous work^15^ but does not significantly change the results. The resulting probability density function (PDF) for possible event locations forms a diffuse spheroidal shell-like distribution in space around the detector registering the neutron. The total probability that the event occurs is then summed over all voxels:

5 $$\begin{aligned} P(E) = \sum _i{P(E | H_i)}. \end{aligned}$$

When applying the NGET algorithm in this work we have applied a light-output threshold of 200 keVee (keV electron equivalent) for all interactions. This threshold corresponds to a neutron energy deposition of 1.3 MeV, due to the non-linear conversion of the deposited neutron energy into scintillation light^23^. The threshold was chosen as a compromise in order to minimize the detection of neutrons scattered inside the radioactive waste itself and the surrounding shielding materials, and the loss of neutrons below the detection threshold, taking into account that the maximum of the fission neutron energy spectrum is located around 2 MeV^28^.

The NGET imaging algorithm^15^ applied in this work is based on D’Agostini’s multidimensional unfolding method^29^ and operates iteratively on the accumulated data on $\Delta t_{n \gamma }$ and *L*. The number of coincidences originating from the voxel *i* is estimated as

6 $$\begin{aligned} n_C(H_i) \approx \sum _{j=1}^{n_E}P(H_i|E_j), \end{aligned}$$

where $n_E$ is the total number of events. As a starting point, the voxel intensity distribution is taken to be uniformly distributed in space. We applied a convergence criterion similar to that adopted by Steinberger et al.^10^.

## Results

### Laboratory measurements

We here present results obtained from measurements on a ^252^Cf radioactive source measured in the laboratory in different conditions. The objective of these measurements was to establish the imaging performance in ideal conditions, with minimum shielding, as well as inside a shielding similar to the external shielding present for typical waste drums. All results presented in this work were obtained from the iterative Bayesian unfolding algorithm described above.

The ^252^Cf source had an emission rate of 3.7 x 10^3^ neutrons/s due to spontaneous fission. It was measured either without external shielding (i.e. in air) or placed inside a mockup waste drum assembly. The ^252^Cf material in the source was incorporated into a ceramic cylinder with 4.6 mm diameter and 6 mm height and encapsulated in a double-welded stainless-steel cylinder with 7.8 mm outer diameter and 10 mm height. The source was positioned manually in the field of view of the radiation sensors by attaching it to a thin string. The mockup waste drum assembly consisted of a 2 mm thick stainless-steel waste drum with 460 mm diameter and 625 mm height surrounded by a 50 mm thick concrete cylinder with 500 mm inner diameter. The setup was centered between the detector assemblies as shown in Fig. 1. Data for each source position were collected for two different azimuthal viewing angles in the horizontal plane, differing by $90^{\circ }$. In this way, the source was measured in a multitude of positions inside the mockup waste drum assembly.

**Fig. 2:**
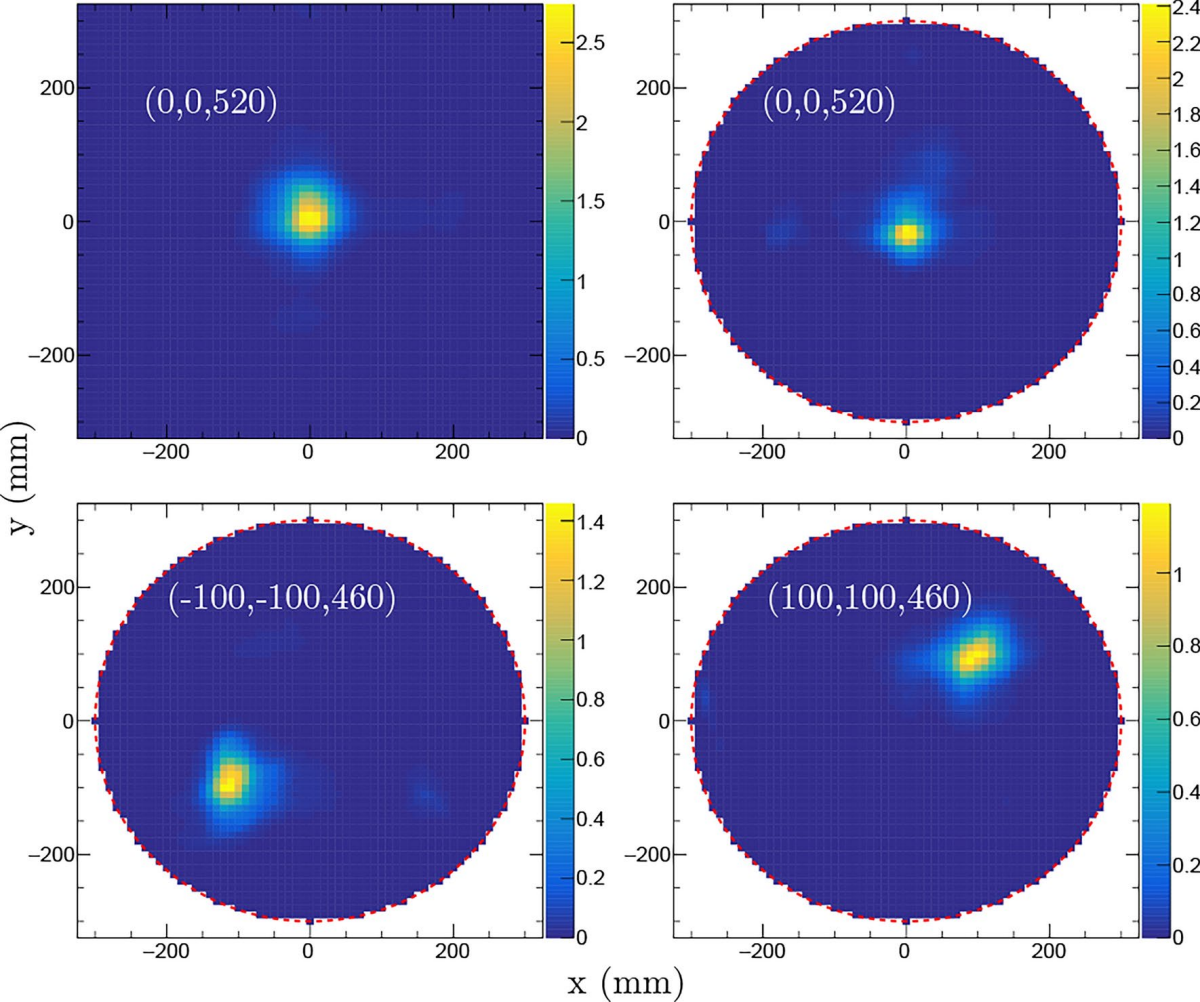
Imaging of the ^252^Cf source described in the text for three different positions (as labeled by their coordinates (*x*, *y*, *z*) in each corresponding panel) using the iterative Bayesian unfolding algorithm. For each position, the source was measured during 15 min at $0^{\circ }$ and 15 min at $90^{\circ }$ with respect to rotation around the vertical *z*-axis through the origin. The images show 2D-projections of the PDF in the $x-y$ plane for the bare source (upper left panel) and for the source placed inside the mockup waste drum assembly (upper right and lower panels). The color scales indicate the number of detected gamma-neutron events per mm^2^.

**Fig. 3:**
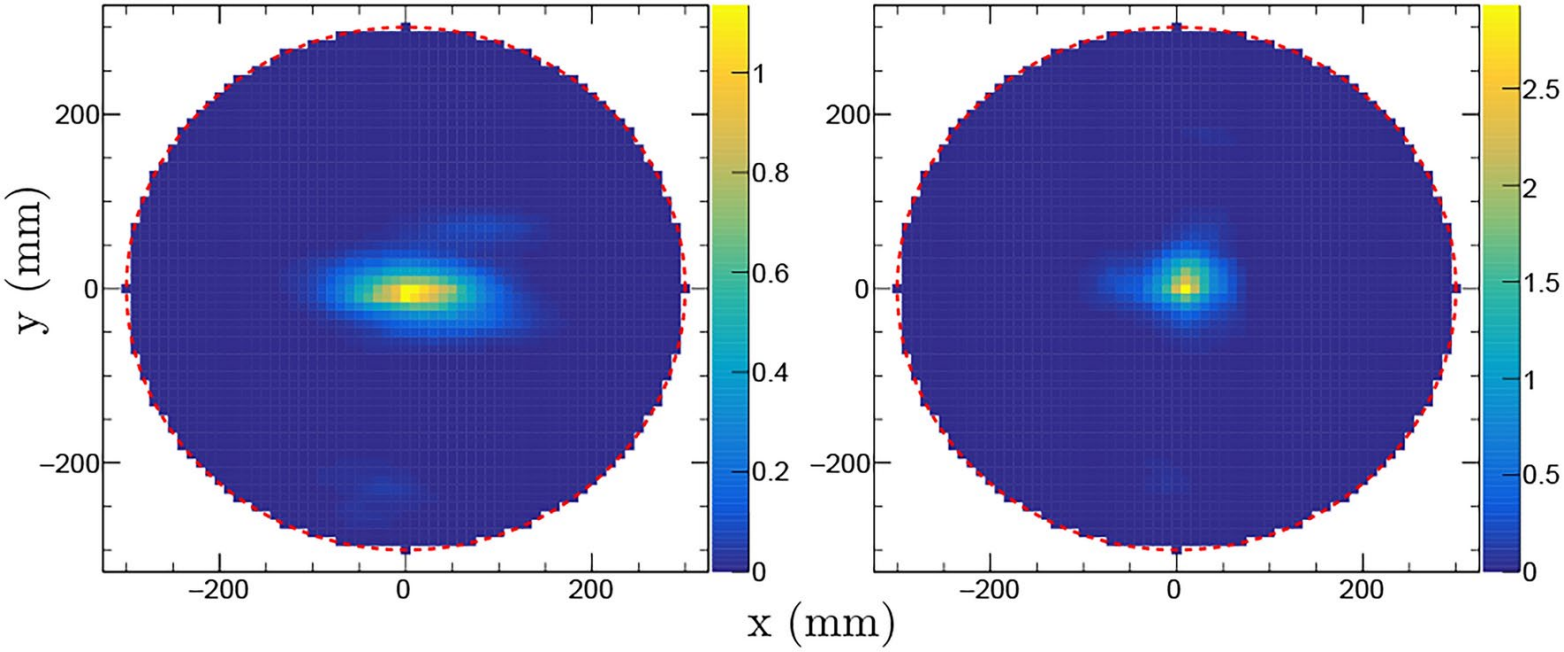
NGET imaging of the ^252^Cf source positioned at (*x*, *y*, *z*) = (0,0,410) mm. The left panel shows results from a 30 min long measurement at $0^{\circ }$ while the image in the right panel is based on combining data from 15 min measurements at $0^{\circ }$ and $90^{\circ }$ with respect to rotation of the mockup waste drum assembly around the vertical *z*-axis. The images are 10 mm thick 2D-projections of the PDF in the $x-y$ plane. The color scales indicate the number of detected events per mm^2^.

Figure 2 shows examples of NGET imaging results based on iterative image reconstruction as described above. The source positions are labeled in mm with their (*x*, *y*, *z*) coordinates relative to the origin, located at the ground level and centered between the detector assemblies. The figure panels show projected two-dimensional (2D) 10 mm thick horizontal slices of the 3D image for each case. The results shown in Fig. 2 were obtained from combining data when the assembly was measured during 15 min at $0^{\circ }$ and 15 min at a $90^{\circ }$ rotation angle. The source was centered between the detector assemblies without any shielding (top left panel) and inside the mockup waste drum assembly for the remaining panels. For the unshielded configuration, the dependence of the imaging performance on the assumed fission neutron kinetic energy spectrum was investigated by replacing the fission spectrum generated by the code FREYA^30^ for ^252^Cf with that for ^240^Pu in the image reconstruction algorithm. The result was a 1 mm or smaller difference in the position determination and less than 10$\%$ deterioration in the image resolution.

In order to investigate the performance of the NGET imaging algorithm for different azimuthal-angle coverage, a single-view measurement was directly compared with a measurement taken at two perpendicular viewing angles with the same total measurement time. Figure 3 shows the results of such measurements with the source placed at (*x*, *y*, *z*) = (0, 0, 410) mm. The image shown in the left panel was obtained from a measurement during 30 min at $0^{\circ }$ while the right panel shows an image obtained when the source was measured during 15 min at $0^{\circ }$ and 15 min at $90^{\circ }$ with respect to rotation of the drum around the vertical *z*-axis. The spatial resolution of the image of the ^252^Cf source taken with one measuring angle is ($\sigma _x$, $\sigma _y$, $\sigma _z$) = (52, 34, 48) mm while the image obtained for the same source position and the same total measurement time at $0^{\circ }$ and $90^{\circ }$ has a spatial resolution of ($\sigma _x$, $\sigma _y$, $\sigma _z$) = (33, 32, 39) mm. Hence, the spatial resolution in, particular, the *x*-direction is improved by the additional measurement angle.

**Fig. 4:**
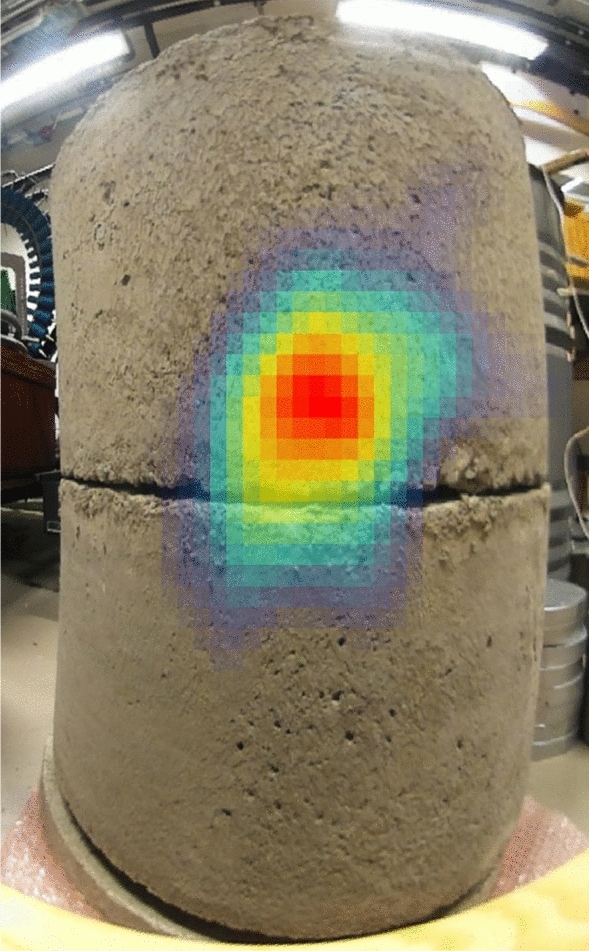
Image of the Cf-source using the commercial gamma-ray imaging system H420 by H3D Inc, USA. The camera was at 20 cm distance from the outer diameter of the cylinder with the source centered in the cylinder at 50 cm distance and was acquired during a 46 minute exposure time. See text for details.

A commercially available gamma imaging system (Model H420 by H3D Inc, Ann Arbor, MI, USA) was also employed in the laboratory setting. This 2D imaging system was able to outline the Cf-source inside the concrete lining and drum with a FWHM of approximately 20 cm at a source distance of 50 cm during 46 minutes of measurement. The automatic parallax correction mode was employed. Imaging based on the 388 keV emission peak, emanating from the radioactive decays of the ^249^Cf isotopic impurity in the source, produced the best results, see Fig. 4. Employing the wide energy collection mode (250 keV to 1.5 MeV) did not improve the localization. The system was operated in Compton imaging mode, since the intensity was not sufficient to allow coded aperture imaging. The 2D Compton image is projected on the fisheye optical image of the concrete cylinder with the camera at a distance of 20 cm from the outer surface of the cylinder. The position of the source as determined by the H3D gamma camera system agreed with that determined by NGET imaging, albeit with a much poorer spatial resolution.

**Fig. 5:**
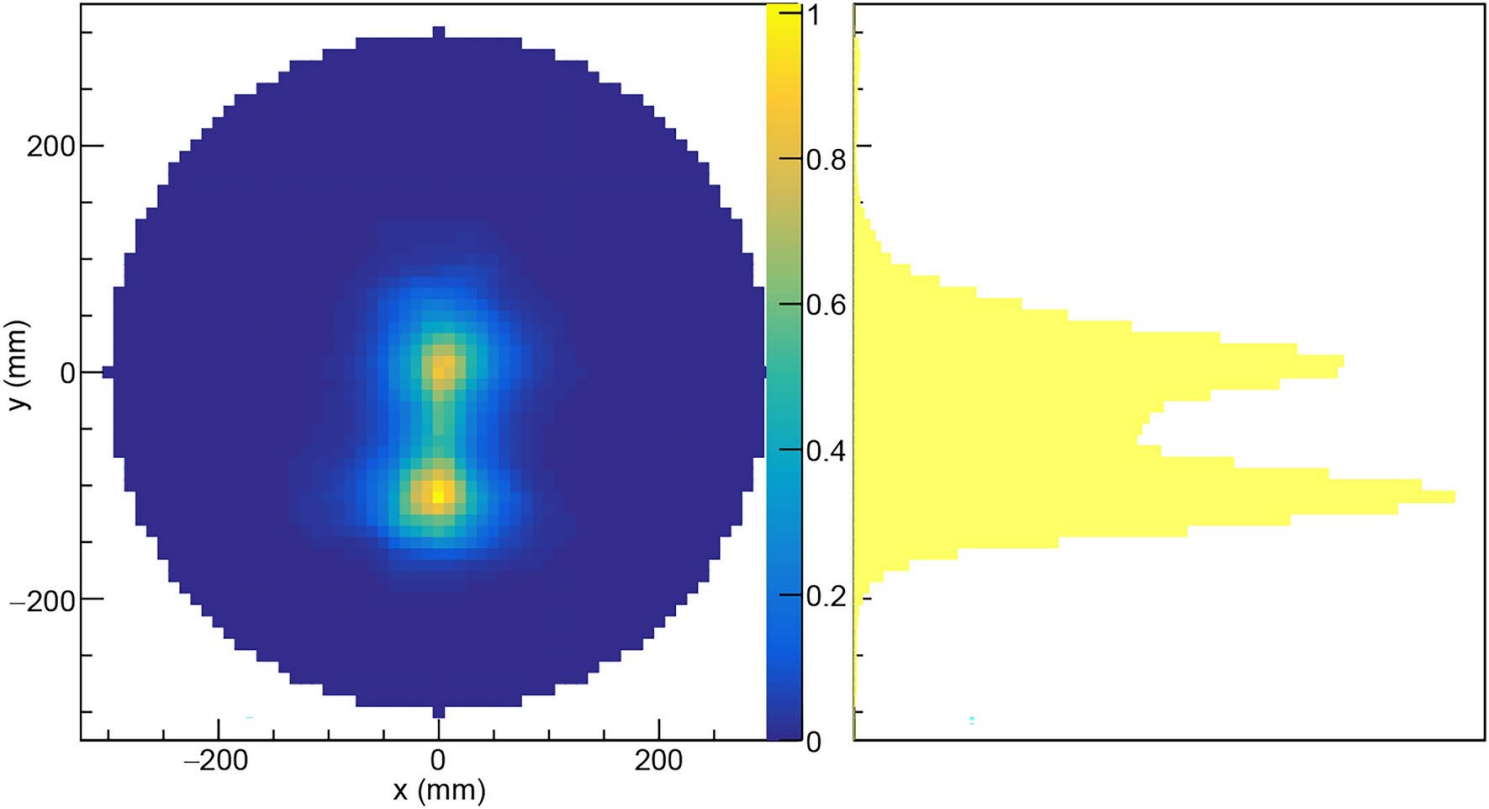
Imaging results for a two-source scenario obtained from measurements on a ^252^Cf sources with $\sim$1 kBq spontaneous fission activity in two positions. The left and right panels show the $x-y$-projection of the PDF and its projection on the y-axis, respectively. The color scale indicates the number of detected events per mm^2^. See text for details.

The NGET imaging algorithm was tested in conditions simulating two spontaneous fission sources present simultaneously inside the mockup waste drum assembly. This was obtained by running the algorithm on a combined data set obtained with the ^252^Cf source placed in two positions, (*x*, *y*, *z*) = (0, − 100, 460) mm and (*x*, *y*, *z*) = (0, 0, 460) mm, i.e. separated by 100 mm. For each position, the measurement time was 15 min at each of the $0^{\circ }$ and $90^{\circ }$ viewing angles. The resulting image obtained from the 2D-projection in the $x-y$ plane is shown in Fig. 5. From the point of view of the NGET imaging algorithm, this is equivalent to imaging two identical sources simultaneously.

**Fig. 6:**
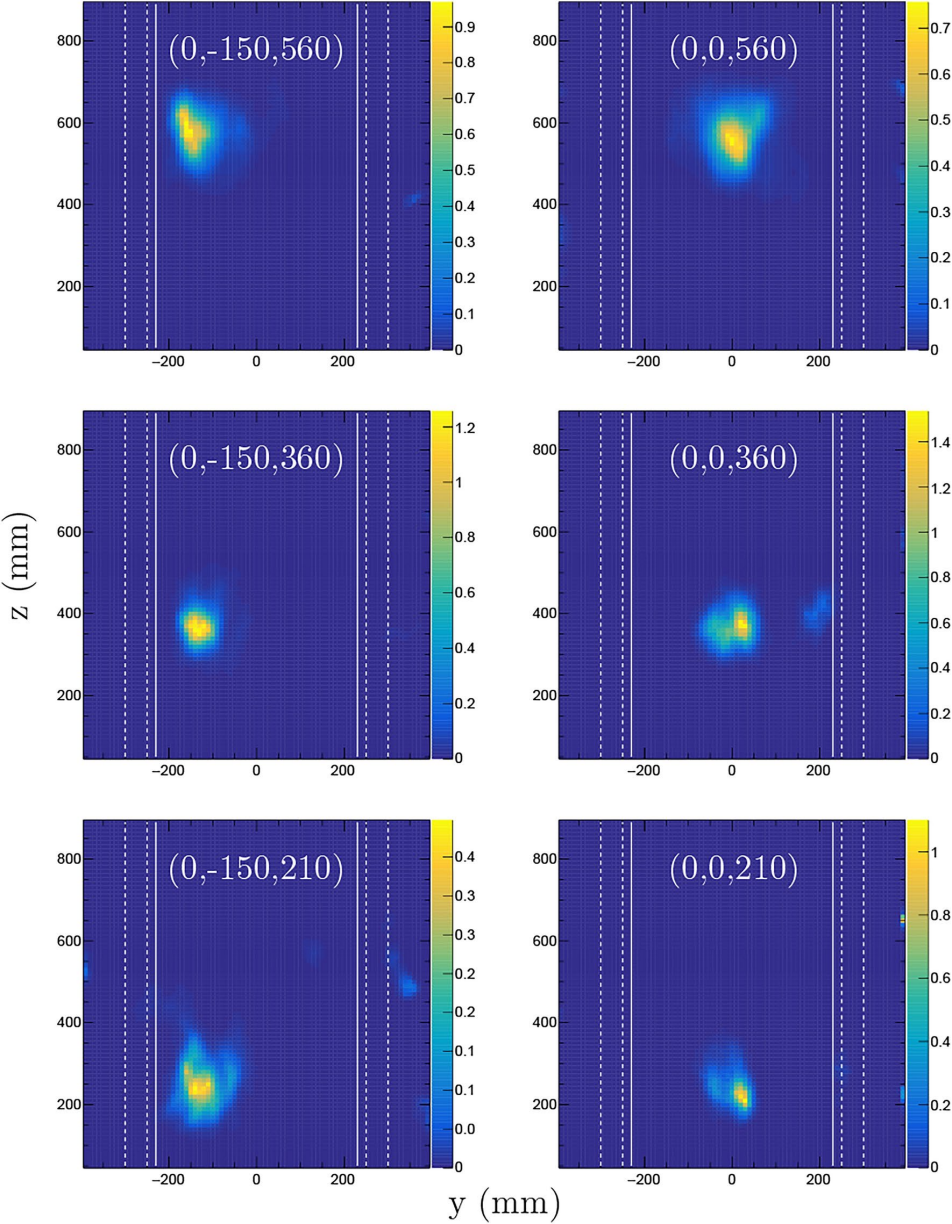
Images of the ^252^Cf source described in the text placed inside the mockup waste drum assembly in six different positions according to the label in each panel. Each source position was measured during 15 min at $0^{\circ }$ and during 15 min after the mockup waste drum assembly had been rotated $90^{\circ }$ in the horizontal $x-y$ plane. The figure shows projections of $y-z$ vertical slices of the 3D PDF taken in the range *x* = [− 5,5] mm. The steel drum wall is indicated by solid white lines. The concrete ring placed around the steel drum is indicated by dashed white lines. The number of detected events per mm^2^ is indicated by the color scales on the right.

**Table 1:** Source positions and their uncertainties deduced from the weighted mean coordinate values ($\mu _x$, $\mu _y$, $\mu _z$), and their standard deviations ($\sigma _x$, $\sigma _y$, $\sigma _z$) of each PDF obtained from the NGET imaging algorithm as shown in Fig. 6. The differences between the deduced and nominal (top row) position coordinates ($\Delta x$, $\Delta y$, $\Delta z$) are also given. The average deviations between the nominal and deduced source position are $\overline{\Delta x}$ = 7 mm, $\overline{\Delta y}$ = 17 mm, and $\overline{\Delta z}$ = 28 mm.

Nom. pos. (*x*, *y*, *z*) mm:	(0,-150,560)	(0,0,560)	(0,-150,360)	(0,0,360)	(0,-150,210)	(0,0,210)
$\mu _x$	11	0.7	3	-4	13	3
$\mu _y$	-129	3	-127	0	-124	8
$\mu _z$	572	570	375	369	258	253
$\sigma _x$	34	37	36	33	35	46
$\sigma _y$	40	40	32	34	39	29
$\sigma _z$	47	53	48	40	54	45
$\Delta x$	11	1	3	4	13	3
$\Delta y$	21	3	23	0	26	8
$\Delta z$	12	10	15	9	48	43

Results from measurements on the ^252^Cf source when placed at different positions inside the mockup waste drum assembly are shown in Fig. 6. Each panel displays the two-dimensional projection of the PDF in the vertical $y-z$ plane. The walls of the inner steel drum and the edges of concrete shielding around the steel drum are represented by white vertical lines and white dashed lines, respectively. The mean position coordinates ($\mu _x$, $\mu _y$, $\mu _z$) and standard deviations ($\sigma _x$, $\sigma _y$, $\sigma _z$) for each PDF and the difference between the nominal and the deduced position are presented in Table 1.

### Test measurements on radioactive waste drums at the Studsvik nuclear facility

**Fig. 7:**
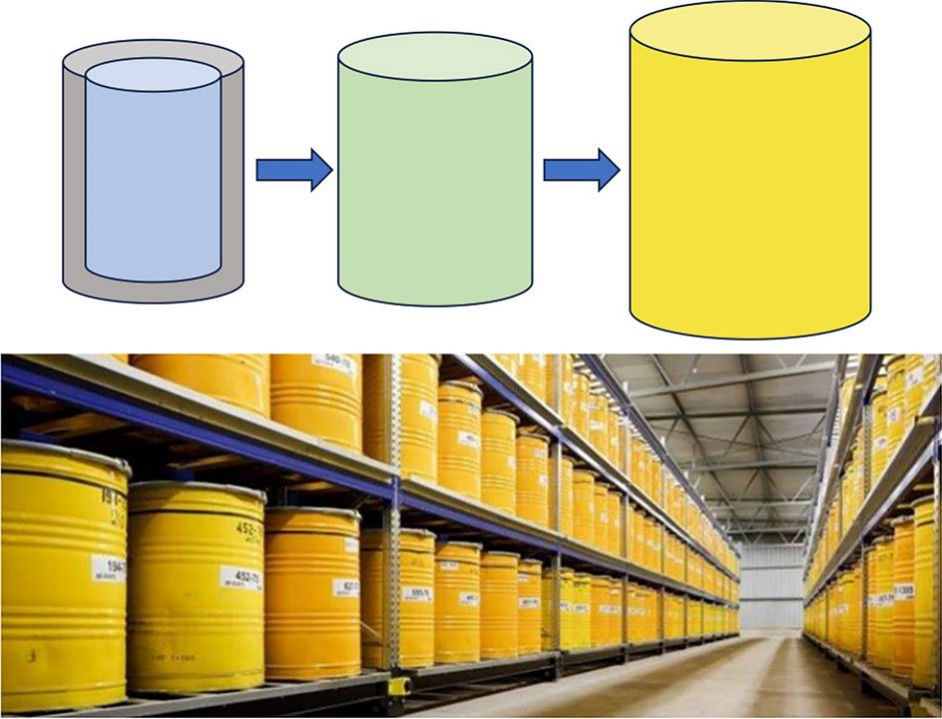
Sketch of typical waste drum configuration (top) and photograph of a part of the “historic” radioactive waste storage facility managed by AB Svafo, Studsvik, Sweden. The radioactive waste (indicated by blue color) is stored in 100 l steel drums or steel mesh baskets and placed in 200 l steel drums (green), with a $\sim$ 50 mm concrete lining (gray) between them. The 200 l drums are over packed in 280 l steel drums (yellow), also shown in the photograph in the lower panel.

We here present results obtained from measurements on waste drums present at the AB Svafo radioactive waste management facility, Studsvik, Sweden. The objective of these measurements was a first test of the NGET performance in real conditions. The drums had been selected as “high interest” cases by AB Svafo from the Swedish legacy waste inventory. The waste was packed in 100 l steel drums or steel mesh baskets. These inner drums were placed in 200 l steel drums and filled with concrete in between. The approximate thickness of the concrete lining is variable but assumed to be at least around 50 mm. Since some of the 200 l drums were corroded, they were previously repacked into 280 l overpack steel drums (Fig. 7). The detailed geometry of the waste drums in real conditions therefore varied somewhat with respect to the mockup waste drum assembly measured in the laboratory.

**Fig. 8:**
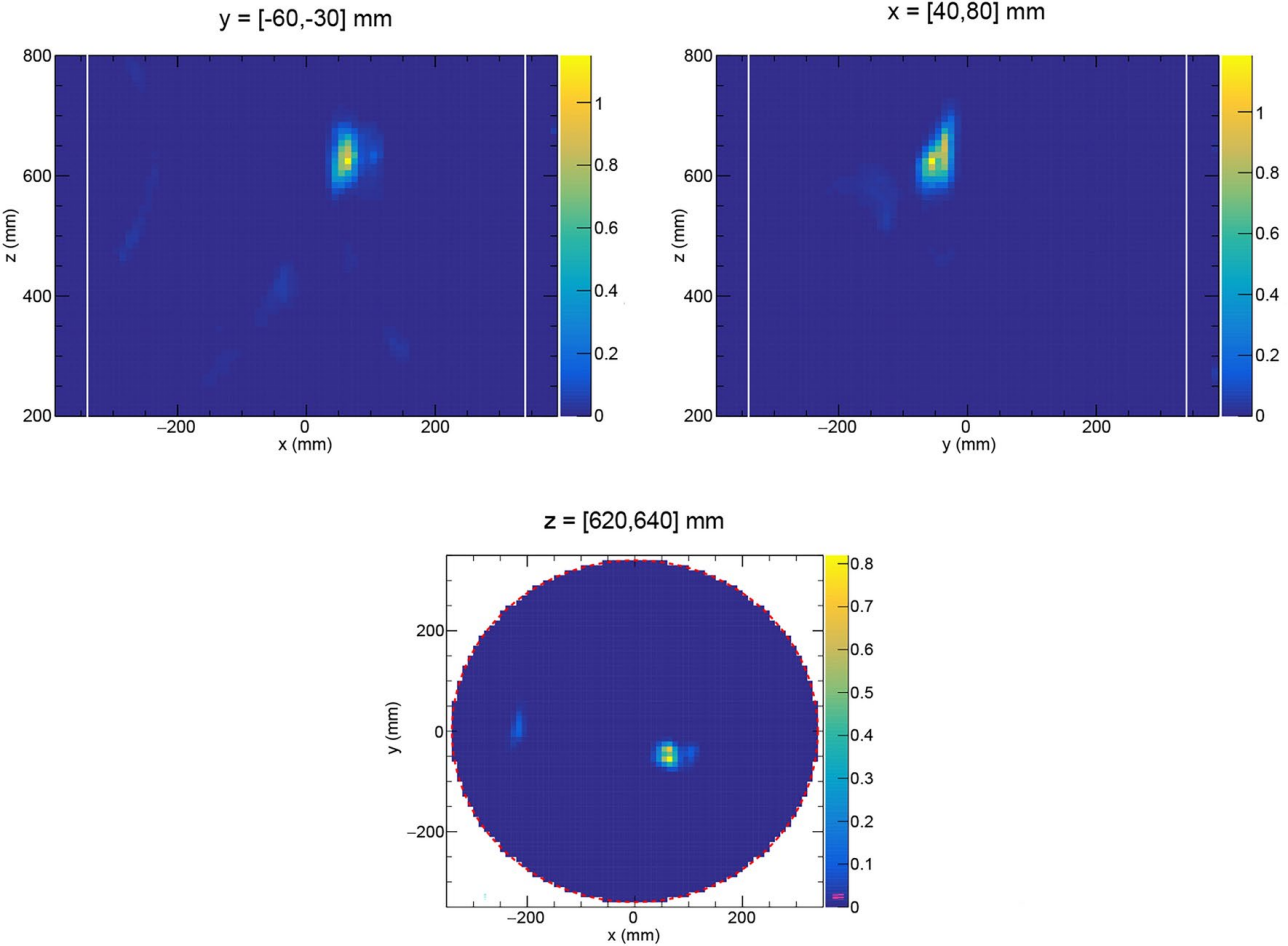
2D-projections of the PDF of the waste drum labeled “FAT 1:1” by AB Svafo. A strong fission source was identified, centered at the position (*x*, *y*, *z*) = (63, − 50, 621) mm. In the vertical projections (top panels), the outer borders of the overpack drum are marked by white lines. The color scales indicate the number of detected gamma-neutron events per mm^2^.

**Fig. 9:**
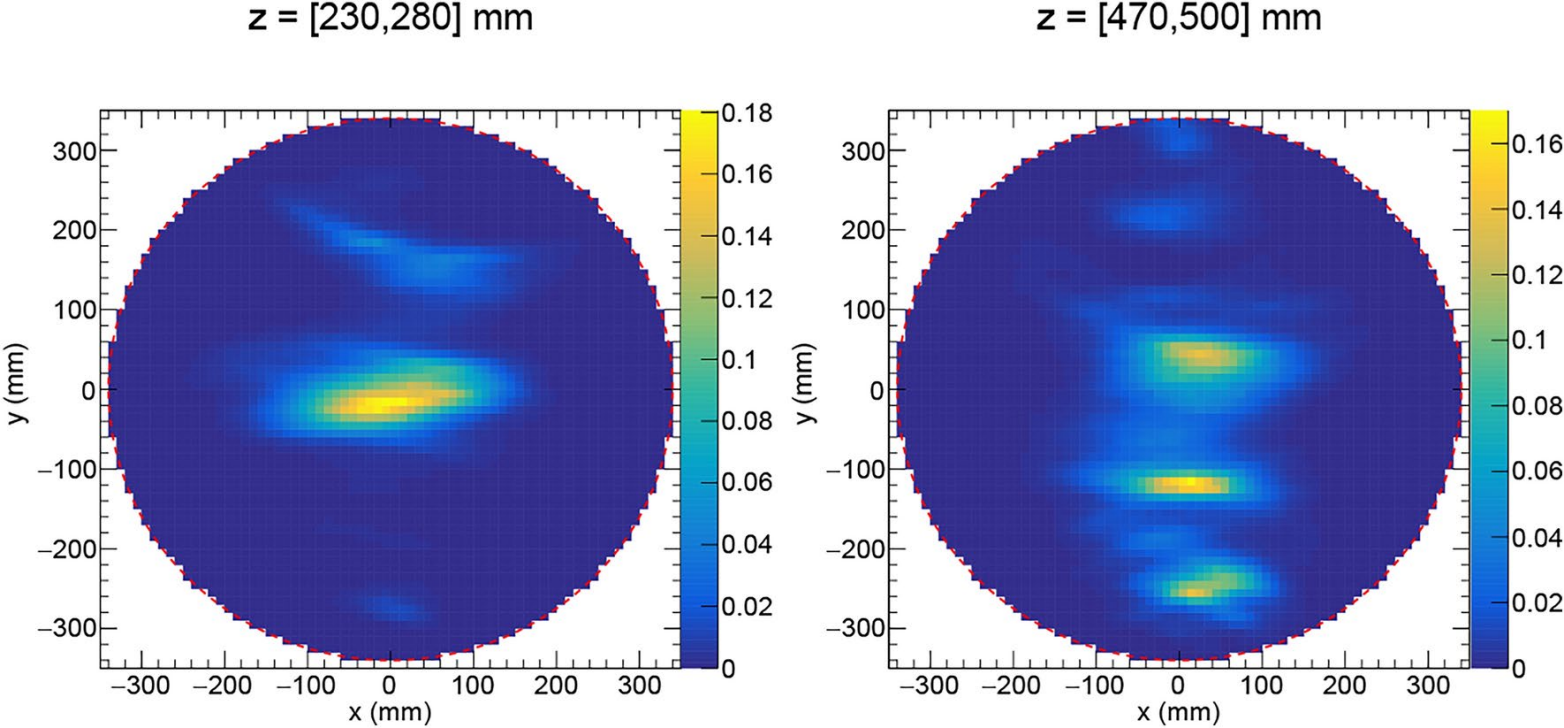
2D-projections of the PDF of the waste drum labeled “FAT 1:2” by AB Svafo. Several fission sources were identified at height *z* = [230, 280] mm and *z* = [470, 500] mm. The color scales indicate the number of detected gamma-neutron events per mm^2^.

No information about the waste matrix composition inside the drums was provided to the measurement team prior to the measurement and subsequent data analysis. Figure 8 shows 2D-projections of the PDF for one of the studied waste drums, labeled “FAT 1:1” by AB Svafo for the purpose of this study. We here show results for data obtained when the drum was measured during 15 min at $0^{\circ }$ and during 15 min with the drum rotated $90^{\circ }$ around the vertical *z*-axis. In the vertical projected images (top panels) the outer border of the overpack drum is indicated by white lines. A fission source was detected at (*x*, *y*, *z*) = (63, − 50, 621) mm, with a spatial distribution ($\sigma _x$, $\sigma _y$, $\sigma _z$) = (19, 25, 43) mm. Comparing these values with the average standard deviation ($\sigma _x$, $\sigma _y$, $\sigma _z$) = (52, 52, 56) mm for the PDF obtained for the ^252^Cf source (Table 1), one may conclude that the source is highly localized and effectively “point-like”. A high-purity germanium (HPGe) detector was used to measure simultaneously gamma-ray emissions from the drum. Characteristic peaks 129.3 keV from ^239^Pu and 160.3 keV from ^240^Pu were identified in the gamma-ray spectrum. The result agrees with results from x-ray imaging previously carried out by AB Svafo on the studied waste drums. A pellet-like object observed in the x-ray images in the upper part of this drum was found to overlap spatially with the NGET image. It is likely that the drum contains remnants of historic actinide separations work. According to the available documentation at AB Svafo, the drum contains in total around 30 g plutonium of unknown isotopic composition.

Figure 9 shows 2D-projections of the PDF for the waste drum labeled “FAT 1:2”. The drum had a relatively low activity and was measured overnight during 16 h 32 min 32 s at one viewing angle. A fission source was identified, centered at the mean position (*x*, *y*, *z*) = (1,14,245) mm in the 3D image of the drum. The source distribution is extended, in particular in the x-direction, due to the single viewing angle. Rotation of the drum during the measurement would increase the resolution in the *x*-direction. Higher in the drum, there are three more fission sources at height *z* = [470,500] mm. Characteristic peaks at 185.7 keV from ^235^U and 1001.0 keV from the ^238^U daughter, ^234m^Pa, were identified in the gamma-ray energy spectrum measured by the HPGe detector. The previous x-ray scanning of the drum showed that it contains a pile of pellet-like objects at the approximate center of the drum and several smaller shielded containers, in good agreement with the NGET imaging results. According to the available documentation at AB Svafo, the drum contains in total 5.6 kg of depleted uranium.

## Discussion

The extreme diversity of radioactive waste, in particular “legacy waste”, poses important challenges for the instruments and methods used for its characterization in order to ensure its safe and effective management. This work demonstrates the feasibility of detecting and imaging actinide materials in shielded drums containing historic radioactive waste using the NGET technique. The technique, is based on measuring gamma-fast neutron correlations from primarily fission reactions using organic scintillator detectors. NGET imaging results have been presented for a ^252^Cf source placed inside a mockup waste drum assembly and on waste drums from the AB Svafo storage facility, Studsvik, Sweden. The laboratory measurement demonstrated that the applied imaging algorithm based on Bayesian inference and iterative image reconstruction only suffered from a slight deterioration in resolution between the bare source measurement and when the source was placed inside a mockup waste drum assembly. The results also demonstrated a good uniformity in the image resolution across the field of view and only small deviations between nominal and measured source positions of less than 30 mm. Furthermore, the imaging performance has been demonstrated to be relatively insensitive to the isotopic differences in the fission neutron spectra. For passive measurements, the decay branching ratio for spontaneous fission and the half life limits the sensitivity for each nuclide of interest.

The NGET 3D imaging technique was compared to a commercially available gamma imaging system. The techniques should be seen as complementary since the 2D gamma imaging system offers considerable advantages for pure gamma-ray imaging such as portability, spectroscopic abilities as well as ease of use. In the current setting the 2D gamma camera, however, achieved a considerably less precise source localization and can be estimated to suffer from a further lower degree of performance for actinides with weaker and less energetic gamma emissions.

From the measurement campaign at AB Svafo, the NGET imaging results were presented for two waste drums. We demonstrated imaging for $\sim$5.6 kg of uranium and $\sim$30 g of plutonium. By comparing imaging results from one measurement angle and the other from two measurement angles ($0^{\circ }$ and $90^{\circ }$), we expect that the imaging resolution with the present detector geometry could be improved by increasing the set of viewing angles by means of controlled movement of the drums during the measurement. In the future, a new waste drum scanning system with controlled movement^31^ will be used for scanning waste containers at AB Svafo. The movement will consist of rotation around the origin in the horizontal plane and a translation along the vertical direction. The prototype scanner system will also perform a high-resolution gamma-ray emission tomography and radionuclide identification using a collimated HPGe detector. Additionally, gamma-ray transmission tomography will be carried out for densitometric purposes.

Although the present study aims at investigating the imaging capabilities of the NGET method as applied to nuclear materials in shielded waste containers, we note that quantitative actinide mass measurements could also be achieved using data from the present experimental setup using neutron multiplicity counting moments (MCM) in combination with standard passive neutron and gamma counting. It is an approach that is commonly used for nuclear materials control in applications relevant for accountability according to international nuclear safeguards. Traditionally, fissile mass estimates are obtained based on mathematical modeling of NMC moments using capture-based detectors such as ^3^He proportional counters^32^. For fast-neutron scatter-based detectors, as in the present application, the formalism needs, however, to be corrected due to neutron scatters between detector elements (cross talk)^33^.

## Data Availability

The datasets generated and analyzed during the current study are available from the corresponding author on reasonable request.
